# Inflammatory Role of ROS-Sensitive AMP-Activated Protein Kinase in the Hypersensitivity of Lung Vagal C Fibers Induced by Intermittent Hypoxia in Rats

**DOI:** 10.3389/fphys.2016.00263

**Published:** 2016-06-27

**Authors:** Chang-Huan Yang, Yan-Jhih Shen, Ching Jung Lai, Yu Ru Kou

**Affiliations:** ^1^Institute of Physiology, School of Medicine, National Yang-Ming UniversityTaipei, Taiwan; ^2^Department of Pharmacology and Toxicology, School of Medicine, Tzu Chi UniversityHualien, Taiwan; ^3^Department of Physiology, Tzu Chi UniversityHualien, Taiwan

**Keywords:** intermittent hypoxia, airway hypersensitivity, lung vagal C fibers, reactive oxygen species, AMP-activated protein kinase

## Abstract

Obstructive sleep apnea (OSA), manifested by airway exposure to intermittent hypoxia (IH), is associated with excess reactive oxygen species (ROS) production in airways, airway inflammation, and hyperreactive airway diseases. The cause-effect relationship for these events remains unclear. We investigated the inflammatory role of ROS-sensitive AMP-activated protein kinase (AMPK) in IH-induced airway hypersensitivity mediated by lung vagal C fibers (LVCFs) in rats. Conscious rats were exposed to room air (RA) or IH with or without treatment with *N*-acetyl-*L*-cysteine (NAC, an antioxidant), Compound C (an AMPK inhibitor), ibuprofen (a cyclooxygenase inhibitor), or their vehicles. Immediately after exposure (24 h), we found that intravenous capsaicin, phenylbiguanide, or α,β-methylene-ATP evoked augmented LVCF-mediated apneic responses and LVCF afferent responses in rats subjected to IH exposure in comparison with those in RA rats. The potentiating effect of IH on LVCF responses decreased at 6 h after and vanished at 12 h after the termination of IH exposure. The potentiating effect of IH on LVCF-mediated apneic and LVCF afferent responses was significantly attenuated by treatment with NAC, compound C, or ibuprofen, but not by their vehicles. Further biochemical analysis revealed that rats exposed to IH displayed increased lung levels of lipid peroxidation (an index of oxidative stress), AMPK phosphorylation (an index of AMPK activation), and prostaglandin E_2_ (a cyclooxygenase metabolite), compared with those exposed to RA. IH-induced increase in lipid peroxidation was considerably suppressed by treatment with NAC but not by compound C or ibuprofen. IH-induced increase in AMPK phosphorylation was totally abolished by NAC or compound C but not by ibuprofen. IH-induced increase in prostaglandin E_2_ was considerably prevented by any of these three inhibitor treatments. The vehicles of these inhibitors exerted no significant effect on the three IH-induced responses. These results suggest that 24-h IH exposure sensitizes LVCFs, leading to an exaggerated reflex and afferent responses to chemical stimulants in rats. Moreover, this IH-induced LVCF sensitization is mediated through a cascade of inflammatory responses in the airways involving increases in ROS, AMPK activation, and cyclooxygenase metabolite release.

## Introduction

Obstructive sleep apnea (OSA), manifested by airway exposure to intermittent hypoxia (IH), is associated with various types of hyperreactive airway diseases such as asthma, chronic cough, and bronchial hyperreactivity in humans (Lin and Lin, 1995; Ciftci et al., 2005; Sundar et al., 2010; Teodorescu et al., 2012). Patients with OSA also display increased oxidative stress and inflammation in the airways (Carpagnano et al., 2008; Petrosyan et al., 2008; Fortuna et al., 2011; Karamanli et al., 2014). In these patients, repetitive periods of upper airway collapse result in cyclic periods of hypoxia/re-oxygenation (Lavie, 2003). This process appears to produce excess reactive oxygen species (ROS) in airways (Prabhakar, 2001), which has been postulated to induce airway inflammation (Carpagnano et al., 2011). Excess ROS and inflammatory mediators are potential contributors for development of hyperreactive airway diseases (Kou et al., 2011); however, the cause-effect relationship for these events in the setting of OSA remains unclear.

Airway hypersensitivity is characterized by augmented sensory and reflex responses to stimuli because of sensitization of lung afferents, particularly lung vagal C-fibers (LVCFs), which have been implicated in the pathogenesis of hyperreactive airway diseases (Widdicombe, 2003; Kou et al., 2011). Accumulating evidence suggests that excess ROS (Tsai et al., 2006, 2009; Taylor-Clark and Undem, 2011; Ruan et al., 2014) and inflammatory mediators, such as cyclooxygenase metabolites (Ho et al., 2000; Lee et al., 2002), may sensitize C-fiber afferents, leading to the development of airway hypersensitivity. Thus far, we recently reported (Shen et al., 2012) that 10 episodes [each consisting of 30 s of hypoxia air exposure followed by 30 s of room air (RA) exposure] of acute IH in rats can induce LVCF-mediated airway hypersensitivity. This immediate hypersensitivity is mediated through excess ROS. However, the duration of IH exposure in the experimental model (Shen et al., 2012) is probably insufficient to induce releases of inflammatory mediators, a situation that differs from that occurring in patients with OSA. Accordingly, an experimental model of IH that can induce a cascade of excess ROS, increased inflammatory mediators, and LVCF hypersensitivity is required for studying the cause-effect relationship and the underlying mechanism.

AMP-activated protein kinase (AMPK) was initially known as a critical regulator of energy homeostasis but was later found to be able to promote increases in inflammatory mediators in cell types other than lung cells (Du et al., 2005; Tang et al., 2007; Chang et al., 2010). In addition, AMPK can be activated by excess ROS (Emerling et al., 2009; Irrcher et al., 2009; Zmijewski et al., 2010). Recently, we have shown that cigarette smoke or toxic smoke increases ROS in the lungs, which in turn activates AMPK, and these events ultimately lead to promotion of lung inflammation (Tang et al., 2011; Perng et al., 2013; Ko et al., 2015). All these observations make AMPK a candidate regulator that contributes to IH-induced LVCF hypersensitivity.

In the light of existing knowledge and the unanswered questions described above, this study was undertaken to investigate (1) whether exposure of rats to IH for 24 h can augment the reflex and afferent responses of LVCFs to chemical stimulants and (2) secondly, whether ROS-sensitive AMPK plays an inflammatory role in IH-induced LVCF hypersensitivity. To achieve these goals, anesthetized, spontaneously breathing rats and anesthetized, artificially ventilated rats were employed with the aim of studying the reflex and afferent responses, respectively. Responses to LVCF stimulants, including capsaicin (Shen et al., 2012), phenylbiguanide (Ruan et al., 2014), and α,β-methylene-ATP (Lin et al., 2013) were measured and served as indices of LVCF reactivity.

## Materials and methods

### Exposure to IH

Male adult Sprague-Dawley rats were used in the experiment. All experimental procedures were approved by the Institutional Animal Care and Use Committee of the Tzu Chi University. Exposure of animals to IH was achieved using previously described methods (Kuo et al., 2011). Briefly, conscious rats were housed in Plexiglas cylindrical chambers (length, 28 cm; diameter, 10 cm; volume, 2.4 liters) with snug-fitting lids. Pure nitrogen was distributed to the chambers by using a timed solenoid valve for 30 s, and the flow was adjusted to reduce ambient FIO_2_ to 2–6% for 2–5 s. Compressed air (45 s) was infused, allowing the gradual return of ambient air to an FIO_2_ of 20.9%. The IH pattern was repeated for 48 times per hour. and the animals were exposed to IH for 24 consecutive h. Rats in RA control group were exposed for 24 h to a similar pattern of gas dynamics in the chamber, with compressed air replacing pure nitrogen.

### General preparations

The method of general preparations has been described in detail previously (Yang et al., 2016). Briefly, at termination of 24 h IH exposure, rats were anesthetized. A segment (~1 cm) of each vagus nerve was carefully isolated from the common carotid artery for later use. A polyethylene catheter was inserted into the jugular vein and advanced until the tip was close to the right atrium for intravenous administration of pharmacological agents. The right femoral artery was cannulated for measurement of arterial blood pressure (ABP) and heart rate (HR). During experiments, supplemental doses of anesthetics were administered to ensure no pain reflexes on pinching the animal tail.

### Measurement of ventilatory responses

For reflex studies, rats were allowed to breathe spontaneously via the tracheal cannula. Respiratory flow was measured using a pneumotachograph (Fleisch 4/0; Richmond, VA, USA) coupled with a differential pressure transducer (Validyne MP45-12) and was integrated to yield tidal volume (V_T_). Tracheal pressure was monitored using a pressure transducer (Validyne MP45-28) via a side tap to the tracheal cannula.

### Perivagal capsaicin treatment

The technique of perivagal capsaicin treatment was adopted from a previous study, and its aim was to selectively block the neural conduction of LVCFs (Kou et al., 1995). In brief, cotton strips soaked in capsaicin solution (250 μg/ml; Sigma) were wrapped around a 2–3 mm segment of the isolated cervical vagus nerves. After 20 min, the cotton strips were removed when the apneic reflex response to an intravenous injection of capsaicin (1 μg/kg) was abolished. The blocking effect of perivagal capsaicin treatment on reflex responses to capsaicin injection was observed to last for 80–120 min (Kou et al., 1995).

### Recording LVCF activity

For electrophysiological studies, afferent activities originating from LVCFs were recorded in open-chest artificially ventilated rats as described in detail previously (Lai and Kou, 1998). Briefly, the right vagus nerve was sectioned. A fine afferent filament was split from the desheathed nerve trunk and placed on a platinum-iridium recording electrode to record afferent nerve activity. The fine filament was further split until afferent activity arising from a single unit was detected. LVCF activity was searched initially by the mild afferent responses to lung hyperinflation (3–4 times V_T_) by occlusion of the expiratory line of the respirator. Furthermore, capsaicin (1 μg/kg), a potent chemical stimulant of LVCFs, was injected as a bolus into the right atrium. Only afferent fibers that showed stimulation within 2 s after the injection were studied. The conduction velocity of afferent fibers of the studied receptors was measured by a previously described method (Ho et al., 2001).

### Intra-Atrial catheter implantation

For the intravenous administration of pharmacological agents, some of the study groups were anesthetized with Zoletil 50 (40 mg/kg, i.p.; Virbac, Carros, France) and implanted with an intra-atrial catheter made of silicone tubing (0.64 mm o.d. × 0.3 mm i.d., 3.5 cm in length; A-M Systems, Sequim, WA, USA) and polyethylene tubing (0.97 mm o.d. × 0.58 mm i.d., 6 cm in length; A-M Systems) through the right external jugular vein, tunneled subcutaneously, exteriorized at the back of the neck, and flame-sealed at 3 days before IH exposure. This implantation was previously described in detail (Pan and Gala, 1985).

### Fluorescence assay to measure lipid peroxidation of lung tissues

Lipid peroxidation was measured using a previously described method (Kuo et al., 2011). In brief, rats were sacrificed, and the lung tissues were immediately extracted and stored at −80°C. For measuring lipid peroxidation present in lung tissue samples, frozen lung tissues were thawed and homogenized in chilled 400 μl chloroform and 200 μl methanol by sonication. After centrifugation, an aliquot of the chloroform, and methanol layer was scanned by using a spectrofluorometer (Perkin-Elmer LS 50B, Waltham, MA, USA). The level of lipid peroxidation was determined by measuring malondialdehyde and its dihydropyridine polymers at 356 nm excitation and 426 nm emission.

### Bronchoalveolar lavage fluid (BALF) preparations

For obtaining BALF samples from rats, saline was intratracheally instilled into the lungs, and then the lungs were lavaged for three times with 3 ml saline containing protease inhibitor cocktail set III (Calbiochem, San Diego, CA, USA). The obtained BALF samples were centrifuged at 1200 × g for 10 min at 4°C and the supernatant of BALF was kept at −80°C for subsequent analysis.

### Measurement of prostaglandin E_2_ (PGE_2_) level by ELISA

PGE_2_ level in BALF supernatants was measured using commercially PGE_2_ high-sensitivity ELISA kit (R&D Systems, Minneapolis, MN, USA) according to the manufacturer's protocol. Optical density was determined on an ELISA reader (Dynex Technologies, UK) at 450 nm and with a wavelength correction at 570 nm. Each well was assayed in duplicate and averaged. With the generation of a four-parameter logarithmic curve, the concentration in ng/ml PGE_2_ in the BALF could be determined.

### Western blot analysis for measuring the expression of AMPK in lung tissues

The method of western blot analysis has been described in detail previously (Yang et al., 2016). Briefly, fresh lung tissues were immediately obtained at 15 min after exposure to RA or IH. Aliquots of tissue lysates were separated on 8% SDS-PAGE and then transblotted onto polyvinylidene fluoride membranes (Merck Millipore Corporation, USA). After being blocked with 5% skim milk, blots were incubated with rabbit primary antibody against rat phospho-AMPK (pAMPK) (1:1000; Cell Signaling, Beverly, MA, USA) or rabbit primary antibody against rat AMPK (1:1000, Cell Signaling), then second goat anti-rabbit IgG antibody (1:1000; Jackson ImmunoResearch, USA). The protein bands were detected by use of an enhanced chemiluminescence kit (PerkinElmer) and quantified by use of an ImageJ processing software.

### Pharmacological agents

To stimulate the LVCFs, capsaicin (1 μg/kg; a TRPV1 receptor agonist) (Shen et al., 2012), phenylbiguanide (8 μg/kg; a 5-HT3 receptor agonist) (Ruan et al., 2014), and α,β-methylene-ATP (15 μg/kg in the reflex studies and 100 μg/kg in the electrophysiological studies; a P2X receptor agonist) (Lin et al., 2013) at 0.1 ml volumes were injected as a bolus into the right atrium. The drugs were first injected into the catheter (dead space = ~0.2 ml) and then flushed into the circulation by an injection of 0.3 ml saline. Treatments of NAC (an antioxidant, 25 mg/kg), Compound C (an AMPK inhibitor, 2 mg/kg), or ibuprofen (a cyclooxygenase inhibitor, 15 mg/kg) were given (volume ~0.35 ml) via the implanted intra-atrial catheter at 15 min prior to IH exposure and at 12 h after the beginning of IH. The vehicle for the stock solution of capsaicin (5 mg/ml) was a solution that contained 10% Tween 80, 10% ethanol, and 80% saline. The vehicle for phenylbiguanide, α,β-methylene-ATP, NAC, and ibuprofen was saline (Vehicle 1). The vehicle for Compound C was prepared solved in 20% dimethyl sulfoxide and then diluted in saline (Vehicle 2). Except for Compound C (Calbiochem, San Diego, CA), all other drugs were purchased from Sigma.

### Experiment design and protocols

A total of 188 rats (weight 320–420 g) were used in this study. In the reflex and electrophysiological studies, 160 rats were evenly divided into 16 study groups to allow five series of experiments to be performed. Each group contained 10 rats, and only one LVCF was studied from one rat in electrophysiological studies. Injection of three types of chemical stimulants (capsaicin, phenylbiguanide, or α,β-methylene-ATP) were separately performed in a counterbalance order. For the baseline respiratory pattern or LVCF fiber activity (FA) to return to its control level, an elapsed time of ~15 min was allowed between any two injections of stimulants. In study series 1, the apneic responses to these three stimulants were studied in rats exposed to RA (*Group 1*) or IH (*Group 2*), with the aim of studying the potentiating effect of IH. Subsequently, the apneic responses to three stimulants were measured after perivagal capsaicin treatment and after bilateral vagotomy to assess the function of LVCFs. In study series 2, the afferent responses of LVCFs to the three stimulants were evaluated in rats immediately upon termination of RA (*Group 3*) or IH (*Group 4*) exposure, as well as at 6 h after (*Group 5*) or at 12 h after (*Group 6*) the end of IH exposure to assess the potentiating effect of IH exposure and its recovery. In study series 3, the apneic reflex responses and afferent responses of LVCFs to the three stimulants were investigated in rats exposed to IH with treatment of NAC (NAC+IH; *Group 7–8*), Compound C (Compound C+IH; *Group 9–10*), ibuprofen (ibuprofen+IH; *Groups 11–12*), Vehicle 1 (the vehicle of NAC or ibuprofen; Vehicle 1+IH, *Groups 13–14*), or Vehicle 2 (the vehicle of Compound C; Vehicle 2+IH; *Groups 15–16*) to assess the functions of ROS, AMPK, and cyclooxygenase metabolites. In study series 4, lung tissues were obtained from *Groups 1, 2, 7, 9, 11, 13*, and *15* (RA or IH in reflex studies) were employed to measure the lung levels of lipid peroxidation, with the aim of assessing IH-induced oxidative stress. In study series 5, BALF were obtained from *Group 3, 4, 8, 10, 12, 14*, and *16* (RA or IH in afferent studies) were employed to measure the concentration of PGE_2_ in BALF. In the study of AMPK activation by IH, lung tissues were obtained from seven additional groups of rats (each *n* = 4) exposed to RA or to IH with or without treatment with NAC, Compound C, ibuprofen, Vehicle 1, or Vehicle 2 to measure the protein levels of pAMPK and AMPK.

### Data analysis and statistics

For the studies of apneic reflex, the baseline T_E_ and V_T_ were calculated on a breath-by-breath basis as the average value in the ten-breath period immediately before the injection of chemical stimulants. To compare the apneic responses evoked by different experimental conditions, the longest T_E_ occurred during the first 20 s after injection of stimulants was divided by the baseline T_E_ to yield the apneic ratio. For the studies of LVCF responses, baseline FA was calculated as the average value over a 10-s interval immediately preceding injection of chemical stimulants. Peak responses were defined as the maximum averaged over a 2-s interval during the 20-s period following the injection of stimulants. In all studies, mean ABP and HR were continuously analyzed at 1-s intervals. Baseline ABP and HR were calculated as the mean value over the 10-s period immediately preceding injection of the stimulant. All physiological signals were analyzed by a computer equipped with an analog-to-digital converter (Gould DASA 4600) and software (BioCybernatics 1.0, Taipei, Taiwan). Data obtained from three or more groups were compared by one-way analysis of variance (ANOVA) or two-way mixed factorial ANOVA, followed by Neuman-Keuls tests when appropriate. A value of *p* < 0.05 was considered significant. All data are presented as means ± SE.

## Results

### Baseline physiological parameters

No significant difference in average body weight between the RA rat (358.5 ± 7.6 g; *n* = 24) and the IH rats (347.1 ± 3.1 g; *n* = 164) was found. Among animals without any drug or vehicle treatment, the mean ABP (107.7 ± 2.7 mmHg) and HR (332.6 ± 7.0 beats/min) of rat treatment with RA (*n* = 20) were not significantly different from those of rat treatment with IH (ABP = 109.7 ± 1.5 mmHg; HR = 335.6 ± 7.2 beats/min) (*n* = 20) when anesthetized. Furthermore, in reflex studies, the baseline f (69.3 ± 3.6 breaths/min), T_E_ (0.51 ± 0.02 s), and V_T_ (1.14 ± 0.09 ml) of the IH rats (*n* = 10) were similar to those of RA rats (*f* = 68.6 ± 2.4 breaths/min; T_E_ = 0.52 ± 0.03 s; V_T_ = 1.16 ± 0.08 ml) (*n* = 10). In electrophysiological studies, a total of 90 LVCFs were measured for responses to the injection of chemical stimulants. The exposure to IH alone caused a slight increase in the baseline FA (0.30 ± 0.03 impulses/s; *n* = 10), but without statistically significant difference in comparison with that of RA exposure (0.07 ± 0.02 impulses/s; *n* = 10), at 6 h after termination of IH exposure (0.09 ± 0.03 impulses/s; *n* = 10), and at 12 h after termination of IH exposure (0.09 ± 0.02 impulses/s; *n* = 10). Among the 90 LVCFs studied, the conduction velocity of 75 fibers was measured (1.12 ± 0.08 m/s; range 0.83–1.69 m/s); the conduction velocity of the remaining 15 fibers was not measured because of the loss of electrophysiological signal. These LVCFs were all localized within the lung structure.

### Role of LVCFs in IH-induced augmented apneic response to chemical stimulants

**Figure 1:**
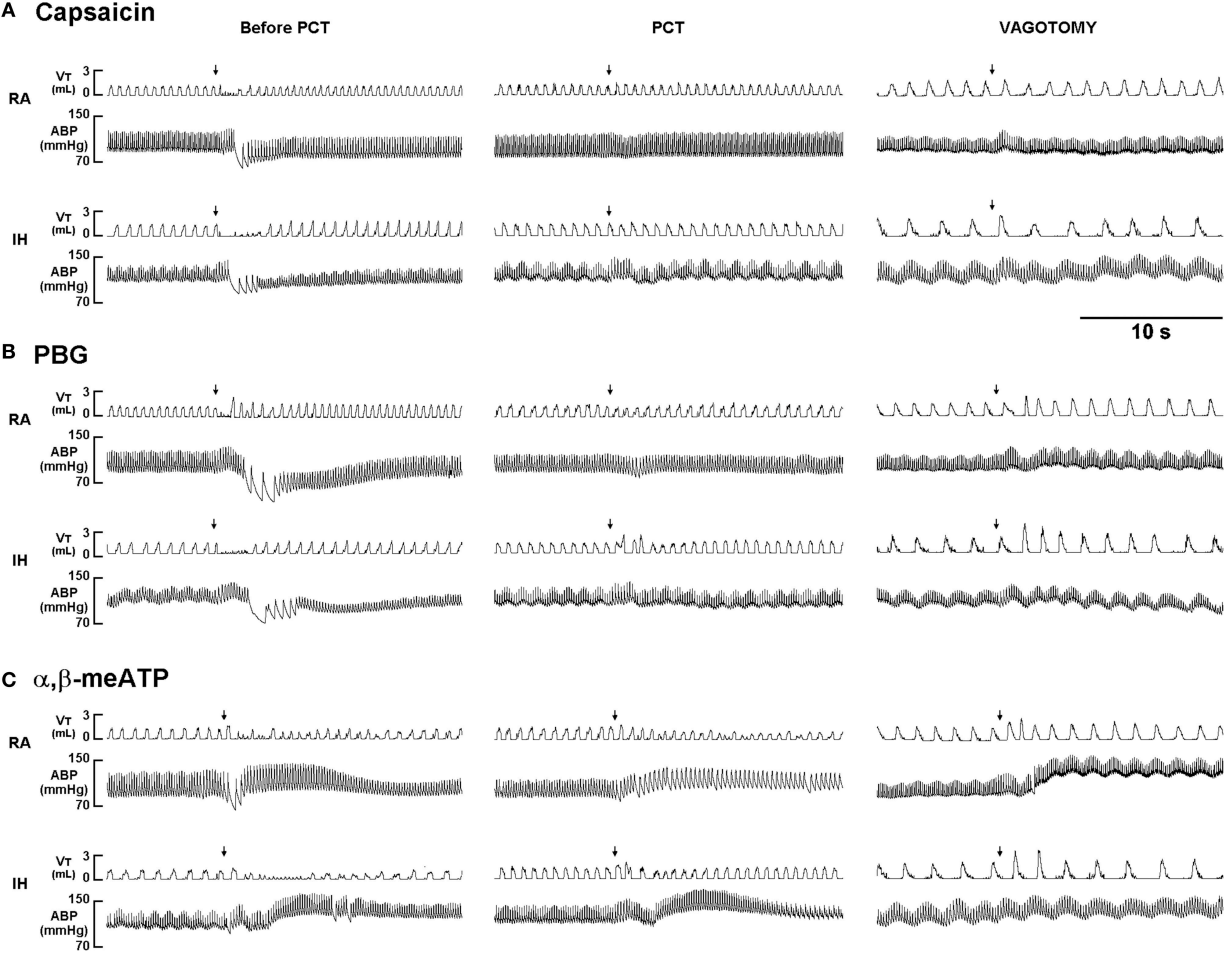
**Ventilatory responses to intravenous injection of three types of stimulants in two rats after exposure to room air or intermittent hypoxia**. The duration of exposure to room air (RA) or intermittent hypoxia (IH) was 24 h. Immediately after the termination of RA or IH exposure, the animal responses to capsaicin (1.0 μg/kg; **A**), phenylbiguanide (PBG, 8 μg/kg; **B**), and α,β-methylene-ATP (α,β-meATP; 15 μg/kg; **C**) were measured in each rat under control conditions, after perivagal capsaicin treatment (PCT; 250 μg/ml), and then after bilateral cervical vagotomy. These stimulants of lung vagal C fibers were injected into the jugular vein as a bolus (0.1 ml volume), as indicated by the arrows. The tip of the injection catheter was close to the right atrium. Approximately 15 min elapsed between any two injections. V_T_, tidal volume; ABP, arterial blood pressure.

**Figure 2:**
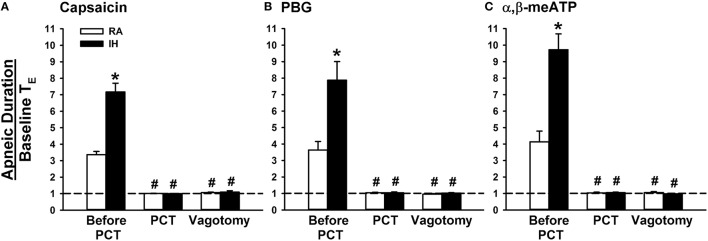
**Role of lung vagal C fibers in intermittent hypoxia-induced augmented apneic responses to stimulants in rats**. Apneic responses to intravenous capsaicin **(A)**, phenylbiguanide (PBG; **B**), and α,β-methylene-ATP (α,β-meATP; **C**) were measured in a counterbalance order immediately after the termination of 24-h exposure to room air (RA) or intermittent hypoxia (IH) in two groups of rats. For each rat, responses to these stimulants were measured during control conditions, after perivagal capsaicin treatment (PCT), and then after vagotomy. The longest expiratory duration (T_E_) occurring during the first 20 s after stimulant injection was divided by the baseline T_E_ to yield the apneic ratio. Baseline T_E_ was calculated as the average of more than 10 consecutive breaths immediately before injections. Horizontal dashed lines indicate an apneic ratio of 1 (no response). *p < 0.05 compared with responses in the RA group under the same experimental condition; ^#^*p* < 0.05 compared with control responses in the same group. Data in each group are means ± SE of 10 rats. See the legend in Figure 1 for further explanation.

When RA rats were investigated, capsaicin injection induced a mild inhibitory effect on breathing, which in turn led to apnea appearing with the prolongation of T_E_ (Figure 1A). Notably, the prolonged T_E_ evoked by the same dose of capsaicin was considerably augmented among IH rats (Figure 1A). As a group, the average apneic response to capsaicin among IH rats was significantly greater than that among RA rats (Figure 2A). Similar results were obtained when phenylbiguanide (Figures 1B, 2B) and α,β-methylene-ATP (Figures 1C, 2C) were used individually as chemical stimulants. Further analysis revealed that two procedures that block the neural conduction of LVCFs (Lin et al., 2013), perivagal capsaicin treatment or bilateral vagotomy, completely abolished apneic responses to intravenous capsaicin, phenylbiguanide, and α,β-methylene-ATP in both RA and IH rats (Figures 1, 2).

### The potentiating effect of IH on afferent responses of LVCFs to chemical stimulants

**Figure 3:**
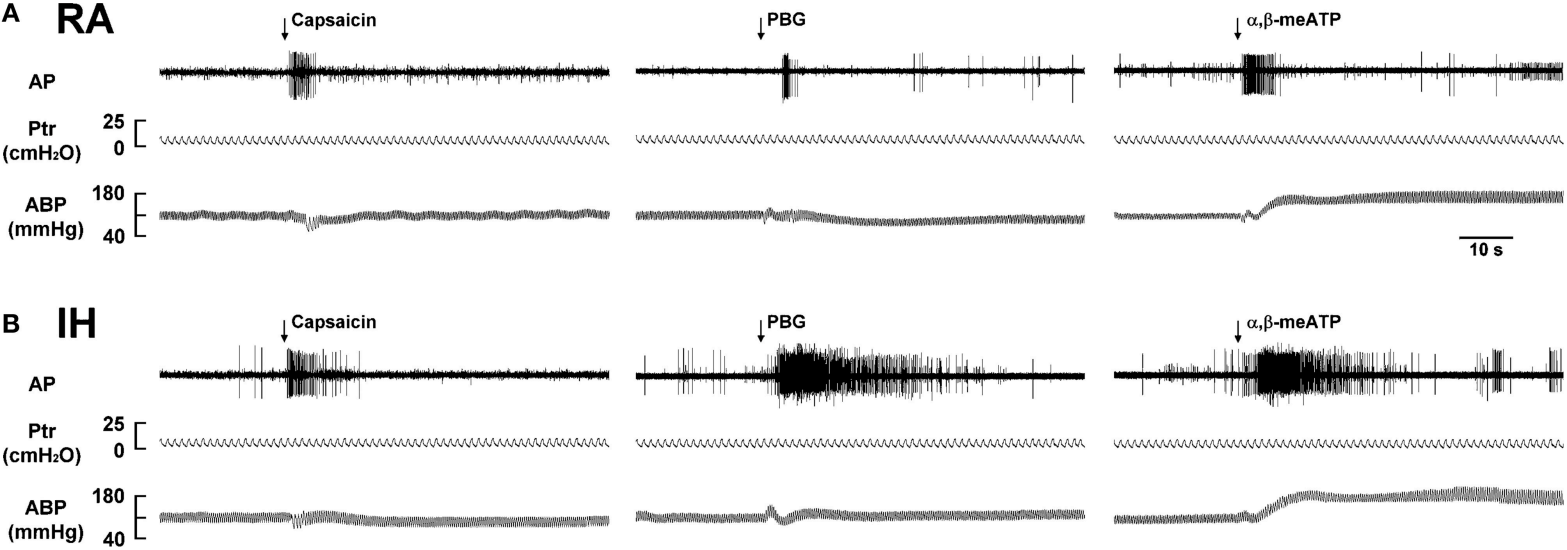
**Responses of lung vagal C fibers to intravenous injection of three types of stimulants in two rats after exposure to room air or intermittent hypoxia**. Duration of exposure to room air (RA; **A**) or intermittent hypoxia (IH; **B**) was 24 h. Immediately after the termination of RA or IH exposure, afferent responses to intravenous capsaicin, phenylbiguanide (PBG), and α,β-methylene-ATP (α,β-meATP) were measured. These stimulants were injected into the jugular vein as a bolus (0.1 ml volume) as indicated by arrows. Approximately 15 min elapsed between any two injections. AP, action potential; P_tr_, tracheal pressure; ABP, arterial blood pressure. See the legend in Figure 1 for further explanation.

**Figure 4:**
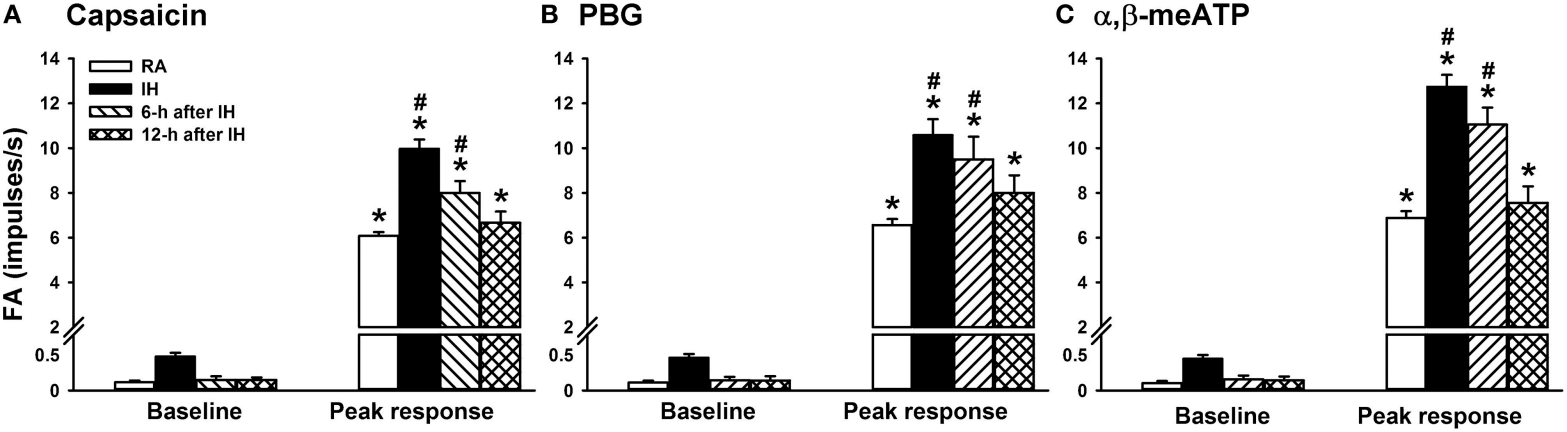
**Sensitizing effect of intermittent hypoxia on the afferent responses of lung vagal C fibers to intravenous stimulants in rats**. One group of rats was subjected 24 h of exposure to room air (RA), whereas the other three groups were subjected to 24-h exposure to intermittent hypoxia (IH). The RA group and one IH group were subjected to experiments immediately after exposure was terminated. The remaining two IH groups were subjected to experiments at 6 or 12 h after the termination of exposure. The afferent responses of rats to intravenous capsaicin **(A)**, phenylbiguanide (PBG; **B**), and α,β-methylene-ATP (α,β-meATP; **C**) were measured in a counterbalance order. Baseline FA was calculated as the value averaged over 10-s intervals before stimulation. The peak response as the maximum averaged over 2-s intervals after stimulation. Data in each group are presented as means ± SE of 10 fibers recorded from 10 rats. *p < 0.05 compared with the baseline in the same group;^#^*p* < 0.05 compared with the peak response in the RA group. See the legend of Figure 1 for further explanation.

We then measured the activity of LVCFs evoked by chemical stimulants to study afferent responses. In RA rats, intravenous capsaicin, phenylbiguanide, or α,β-methylene-ATP all evoked an abrupt burst of discharge (Figure 3A). Afferent response to any one of these stimulants was markedly augmented among IH rats (Figure 3B). As a group, the mean peak responses to capsaicin (Figure 4A), phenylbiguanide (Figure 4B), and α,β-methylene-ATP (Figure 4C) among IH rats were all significantly greater than those among the RA rats. The potentiating effect of IH decreased at 6 h after and vanished at 12 h after the termination of IH exposure (Figure 4).

### Role of ROS, AMPK, and cyclooxygenase metabolites in the potentiating effect of IH

**Figure 5:**
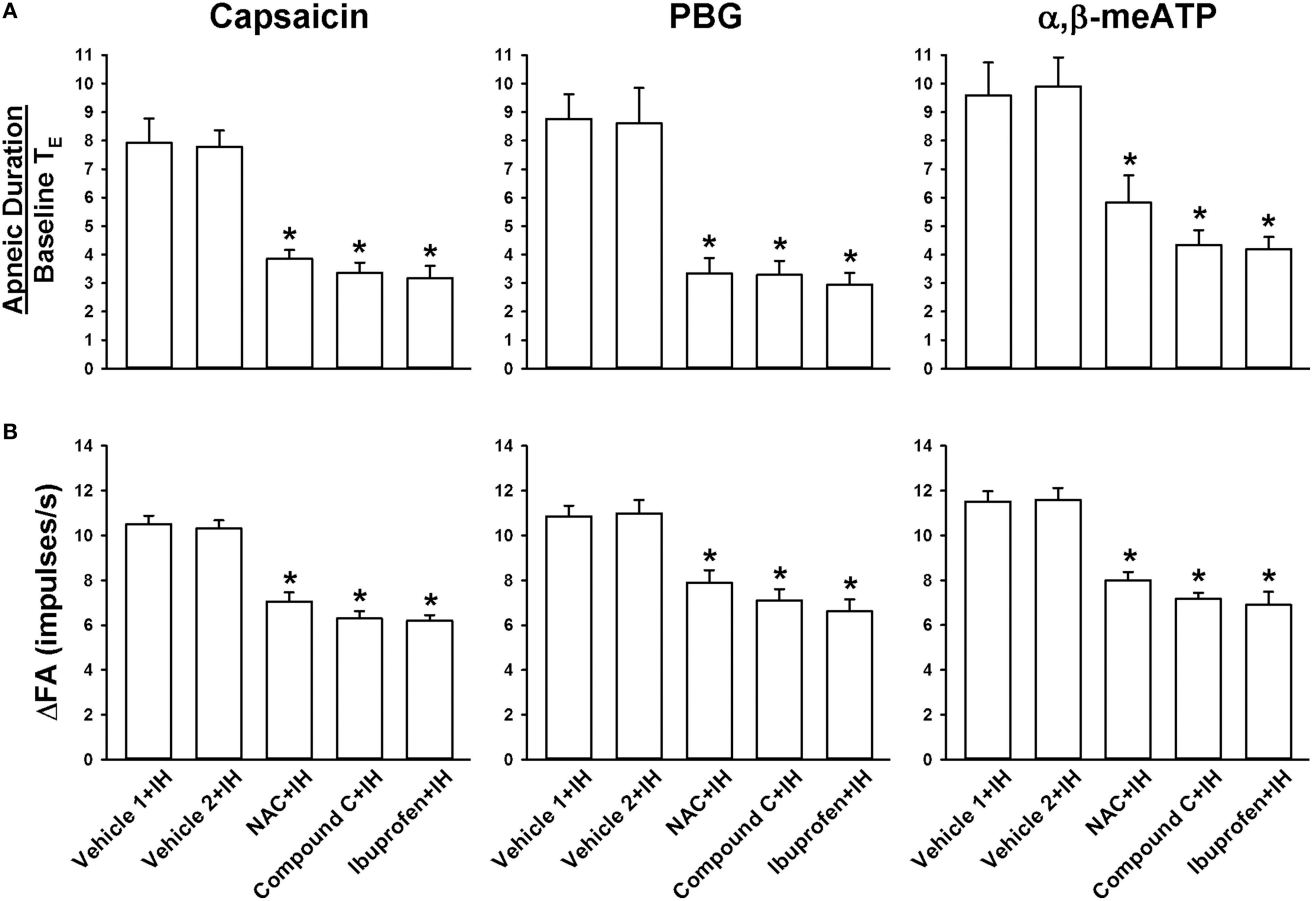
**Roles of ROS, AMPK, and cyclooxygenase metabolites in the intermittent hypoxia-induced potentiating effect of apneic responses and afferent responses to stimulants in rats**. Apneic responses **(A)** and afferent responses **(B)** to intravenous capsaicin, phenylbiguanide (PBG), and α,β-methylene-ATP (α,β-meATP) were measured in a counterbalance order from each rat immediately after the termination of 24-h exposure to intermittent hypoxia (IH) in five groups of rats. In each rat, two treatments of *N*-acetyl-*L*-cysteine (NAC, an antioxidant, 25 mg/kg; NAC+IH), Compound C (an AMPK inhibitor, 2 mg/kg; Compound C+IH), ibuprofen (a cyclooxygenase inhibitor, 15 mg/kg; ibuprofen+IH), Vehicle 1 (the vehicle of NAC or ibuprofen; Vehicle 1+IH), or Vehicle 2 (the vehicle of Compound C; Vehicle 2+IH) were administered intravenously at 15 min prior to IH exposure and at 12 h after the beginning of IH. *p < 0.05 compared with IH with the corresponding vehicle treatment. Data in each group are means ± SE of 10 rats. See legends in Figures 1, 2 for further explanation.

We further employed an antioxidant (NAC), an AMPK inhibitor (Compound C), and a cyclooxygenase inhibitor (ibuprofen) to assess the suppressive effects on the potentiating effect of IH. In reflex studies (Figure 5A), the augmented apneic responses to capsaicin, phenylbiguanide, and α,β-methylene-ATP observed among the IH rats with Vehicle 1 or Vehicle 2 treatment were found to be significantly reduced among IH rats treated with either NAC, Compound C, or ibuprofen. Consistently in the electrophysiological studies (Figure 5B), increases in LVCF response (ΔFA = peak FA − baseline FA) to capsaicin, phenylbiguanide, and α,β-methylene-ATP observed among the IH rats with Vehicle 1 or Vehicle 2 treatment were also found to be significantly attenuated among the IH rats treated with either NAC, Compound C, or ibuprofen.

### IH-induced lung levels of oxidative stress, AMPK phosphorylation, and PGE_2_

**Figure 6:**
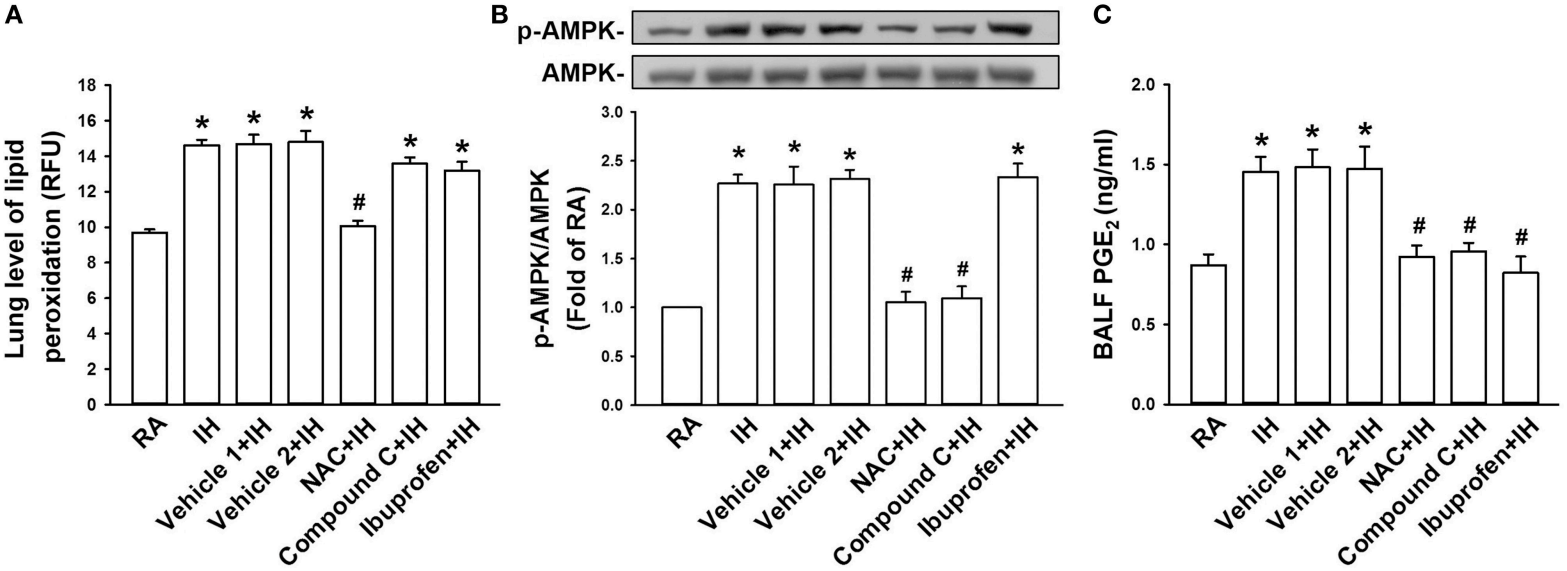
**Exposure of IH increases lung levels of oxidative stress, AMPK phosphorylation, and PGE_2_ in rats**. Samples of lung tissues or bronchoalveolar lavage fluid (BALF) were obtained immediately after the termination of 24-h exposure to room air (RA) or intermittent hypoxia (IH) in seven groups of rats with or without treatment of *N*-acetyl-*L*-cysteine (NAC, an antioxidant; NAC+IH), Compound C (an AMPK inhibitor; Compound C+IH), ibuprofen (a cyclooxygenase inhibitor; Ibuprofen+IH), Vehicle 1 (the vehicle of NAC or ibuprofen; Vehicle 1+IH), or Vehicle 2 (the vehicle of Compound C; Vehicle 2+IH). **(A)** Levels of lipid peroxidation in lung tissues were measured by fluorescence assay to assess oxidative stress. RFU, relative fluorescence unit. **(B)** Levels of the protein expression of phospho-AMPK and AMPK in lung tissues were analyzed by Western blot to assess the activation of AMPK. **(C)** Levels of PGE_2_ in samples of BALF were analyzed by ELISA kit. *p < 0.05 compared with RA group. ^#^*p* < 0.05 compared with IH with corresponding vehicle treatment. Data in each group are means ± SE of 10 rats in panels **(A,C)** and of four rats in panel **(B)**. See legend in Figure 5 for further explanation.

We finally attempted to determine the cascade of oxidative stress, phosphorylation of AMPK, and release of PGE_2_ induced by IH. The levels of lipid peroxidation (Figure 6A) and AMPK phosphorylation (Figure 6B) in lung tissues as well as PGE_2_ in BALF (Figure 6C) were found to be increased in IH rats compared with those in RA rats. IH-induced increases in lipid peroxidation were considerably suppressed by treatment with NAC but not by Compound C or ibuprofen (Figure 6A). IH-induced increases in AMPK phosphorylation was totally abolished by NAC or Compound C but not by ibuprofen (Figure 6B). IH-induced increases in PGE_2_ were largely prevented by any of the three inhibitor treatments (Figure 6C). The vehicles of these inhibitors did not significantly affect the three IH-induced responses (Figure 6).

## Discussion

The results of this study demonstrate that, immediately after exposure, rats treated with 24-h IH displayed augmented apneic responses to intravenous administration of three different LVCF stimulants as compared with rats treated with 24-h RA exposure (Figures 1, 2). The augmented apneic responses to these three stimulants were abolished by perivagal capsaicin treatment or vagotomy (Figures 1, 2), suggesting that these responses were reflex consequences mediated through LVCFs. Indeed, our electrophysiological studies revealed that afferent responses of LVCFs to the stimulants were also augmented among IH rats as compared with those among RA rats (Figures 3, 4). More importantly, the potentiating effects of IH exposure on LVCF-mediated apneic responses or afferent responses to these stimulants were markedly attenuated by treatment with NAC, Compound C, or ibuprofen (Figure 5), indicating the contributions of ROS, AMPK activation, and cyclooxygenase metabolites to the sensitization of LVCFs. This notion is strongly supported by the results from our biochemical measurements, which indicate that IH exposure increased lung levels of lipid peroxidation (an index of oxidative stress), AMPK phosphorylation (an index of AMPK activation), and PGE_2_ (a cyclooxygenase metabolite) (Figure 6). Further analysis of results from inhibitor treatments (Figure 6) revealed that IH increased ROS, which in turn activated AMPK. These events ultimately led to promotion of PGE_2_ production in the airways. Collectively, these results suggest that 24-h IH exposure sensitizes LVCFs, leading to an exaggerated reflex and afferent responses to chemical stimulants in rats. This sensitizing effect is mediated at least in part through a cascade of inflammatory responses in the airways involving increases in ROS, an activation of AMPK, and releases of cyclooxygenase metabolites.

Not surprisingly, excess ROS plays an important role in the IH-induced sensitization of LVCFs. Similar contributions are also found in the development of LVCF hypersensitivity induced by acute IH (Shen et al., 2012) or 14-day chronic IH (Yang et al., 2016), as well as the development of several other cardiorespiratory responses induced by IH (Nisbet et al., 2009; Edge et al., 2012; Schulz et al., 2014). IH produces cyclic periods of hypoxia/re-oxygenation, which seems to promote ROS production in the airways (Prabhakar, 2001). Although ROS sources were not identified in this study, NADPH oxidase, a superoxide anion-generating enzyme, has been suggested as the primary enzyme system for IH-induced ROS production (Nisbet et al., 2009; Edge et al., 2012; Schulz et al., 2014; Yang et al., 2016). In this study, the sensitizing effect of IH on LVCFs persisted at 6 h after and vanished at 12 h after the termination of IH exposure. ROS may directly or indirectly sensitize LVCFs (Taylor-Clark and Undem, 2011; Ruan et al., 2014). In a recent study (Yang et al., 2016), we reported that 14-day exposure of IH can also sensitize LVCFs in rats and that this sensitization is mediated via ROS generated by NADPH oxidase. However, whether the function of ROS is a direct effect or secondary to the release of ROS-induced mediators was not determined in that study. Considering the short half-life of ROS, it is very plausible that mediators other than ROS are involved in this sensitizing effect. This possibility is supported by our finding regarding the involvement of cyclooxygenase metabolites. More importantly, we provide evidence suggesting that the production of cyclooxygenase metabolites are due to excess ROS induced by IH. Exogenous PGE_2_ has been shown to enhance the stimulatory effects of chemical stimulants on LVCFs *in vivo* (Ho et al., 2000; Lee et al., 2002) and *in vitro* (Kwong and Lee, 2002). It is likely that airway epithelial cells are the major source of production of ROS and cyclooxygenase metabolites because the lung epithelium is a primary target for direct insult by IH. In fact, patients with OSA are known to exhibit augmented productions of cyclooxygenase metabolites in the blood (Mejza et al., 2016) or the airways (Goldbart et al., 2006).

One compelling finding in this study is the inflammatory function of ROS-sensitive AMPK. AMPK was initially known as a critical regulator of energy homeostasis (Steinberg and Kemp, 2009; Li and Keaney, 2010). AMPK is activated by an increase in intracellular AMP/ATP ratio under several physiological or pathological conditions, such as glucose deprivation, strenuous exercise, or ischemia that depletes cellular energy (Steinberg and Kemp, 2009; Li and Keaney, 2010). Activation of AMPK can limit further ATP depletion and promote compensatory changes to restore cellular ATP levels (Steinberg and Kemp, 2009; Li and Keaney, 2010). However, AMPK was later found to perform multiple functions that are independent of energy regulation and thus has been implicated in several diseases (Steinberg and Kemp, 2009; Li and Keaney, 2010). Increasing *in vitro* evidence suggests that AMPK can be activated by excess ROS in various cell types, including lung cells (Emerling et al., 2009; Irrcher et al., 2009; Zmijewski et al., 2010; Tang et al., 2011; Perng et al., 2013; Ko et al., 2015). In addition, activation of AMPK by pharmacological activators or inflammatory stimuli can promote the production of several inflammatory mediators, including cyclooxygenase metabolites in different cell types (Du et al., 2005; Tang et al., 2007; Chang et al., 2010). Furthermore, exposure of mice to cigarette smoke (Tang et al., 2011; Ko et al., 2015) or toxic smoke (Perng et al., 2013), which are two types of potent inhaled oxidants, promotes AMPK phosphorylation and inflammation in the lungs; both consequences can be reduced by treatment with Compound C. Meanwhile, our observations are in good agreement with those *in vivo* and *in vitro* findings reported previously. This study appears to be the first to report the inflammatory function of ROS-sensitive AMPK in the IH-induced sensitization of LVCFs.

The mechanism by which IH exposure markedly enhances the sensitivity of LVCFs to different chemical stimulants remains unclear. Recent studies have reported that excess ROS may sensitize airway vagal C fibers, in turn leading to the development of airway hypersensitivity (Tsai et al., 2006, 2009; Shen et al., 2012; Ruan et al., 2014). Thus, ROS are likely potential mediators responsible for IH-induced consequences observed in this study. However, judging from our result regarding the suppressive effect of ibuprofen (Figure 5), cyclooxygenase metabolites that are produced after the activation of AMPK by excess ROS appear to be the major mediators. Capsaicin, phenylbiguanide, and α,β-methylene-ATP are agonists of TRPV1, 5-HT3, and P2X receptors, respectively, which are located at the terminals of LVCFs (Lin et al., 2009, 2013). These types of receptors are ligand-gated ion channels that are mainly permeable to Ca^2+^ (Taylor-Clark and Undem, 2006, 2011). Thus, it appears that IH-induced ROS and cyclooxygenase metabolites may produce nonspecific increases in the electrical excitability of LVCFs regardless of stimulation by different types of agonists. Alternatively, the activation of the three types of receptors by IH-induced ROS and cyclooxygenase metabolites may possibly be promoted by a common cellular mechanism that sensitizes the functioning of ionotropic receptors. In fact, previous studies have shown that exogenous PGE_2_ can nonspecifically enhance the stimulatory effects of different stimulants (capsaicin, lactic acid, and adenosine) on LVCFs in rats (Ho et al., 2000; Lee et al., 2002), and can nonspecifically increase the whole-cell current density elicited by capsaicin and phenylbiguanide in rat lung C neurons (Kwong and Lee, 2002).

Several clinical studies have reported that patients with OSA is associated with asthma (Ciftci et al., 2005; Teodorescu et al., 2012), chronic cough (Sundar et al., 2010), and bronchial hyperreactivity (Lin and Lin, 1995). LVCF hypersensitivity has been implicated in the development of hyperreactive airway diseases (Lee and Pisarri, 2001; Gu et al., 2003). These patients also display increased oxidative stress and inflammation in the airways (Carpagnano et al., 2008; Petrosyan et al., 2008; Fortuna et al., 2011; Karamanli et al., 2014). In these patients, nasal continuous positive airway pressure is a standard therapy to alleviate repetitive episodes of hypoxia/re-oxygenation (Alonso-Fernandez et al., 2009). Whereas nasal continuous positive airway pressure is effective in preventing oxidative stress and inflammation in the airways of patients with OSA (Petrosyan et al., 2008; Carpagnano et al., 2011; Fortuna et al., 2011; Karamanli et al., 2014), this therapy also reduces symtoms of hyperreactive airway diseases (Lin and Lin, 1995; Ciftci et al., 2005; Sundar et al., 2010; Teodorescu et al., 2012). These clinical studies highlight the link among excess ROS, airway inflammation, and hyperreactive airway diseases in this patient population. Herein, we established an experimental model to prove the cause-effect relationship of the three pathophysiological events and identify the underlying mediator mechanisms. We are aware of the fact that OSA involves a chronic IH exposure with longer duration than that in our experimental model. Perhaps, mediator mechanisms underlying the chronic IH-induced hypersensitivity of LVCFs are more complicated than that reported in this study.

In conclusion, the results from the present study demonstrate that 24-h IH exposure sensitizes LVCFs and augments reflex apneic responses to chemical stimulants of LVCFs. This sensitizing effect of IH appears to be mediated through a cascade of inflammatory responses involving oxidative stress, AMPK activation, and releases of cyclooxygenase metabolites in the airways. Our findings may provide novel information for better understanding of the pathogenic mechanisms of OSA-associated hyperreactive airway diseases and potential therapy.

## Author contributions

Conception and design of research: CJL and YRK. Performed experiments: CHY, and YJS. Analysis and interpretation of data: CHY, YJS, CJL, and YRK. Prepared figures: CHY, YJS, CJL, and YRK. Edited and revised manuscript: CHY, YJS, CJL, and YRK.

## Funding

This study was supported by grants MOST 104-2320-B-010-014-MY3 and MOST 103-2320-B-320-002 from Ministry of Science and Technology, Taiwan, and a grant TCIRP101003-01 from Tzu Chi University, Taiwan.

### Conflict of interest statement

The authors declare that the research was conducted in the absence of any commercial or financial relationships that could be construed as a potential conflict of interest.
